# Supplementary material for: Active site specificity profiling datasets of matrix metalloproteinases (MMPs) 1, 2, 3, 7, 8, 9, 12, 13 and 14
Source: Data Brief. 2016 Feb 22;7:299–310. doi: 10.1016/j.dib.2016.02.036 (PMC4777984; doi:10.1016/j.dib.2016.02.036)
Supplement: Supplementary file 10 — Supplementary material [file mmc10.zip › WebPICS_hMMP14_T_1%/P2.html]

 

PICS results


|  |  |
| --- | --- |
| **P2\_A**  66 in 307 sites   21.5 %    effects > 10 perc. pnts.  (vice-versa in brackets)  P2prime\_L: -11.5 (-15.4) |  |
| **P2\_C**  12 in 307 sites   3.9 %    effects > 10 perc. pnts.  (vice-versa in brackets)  P1prime\_Q: 20.4 (17.5) |  |
| **P2\_E**  23 in 307 sites   7.5 %    effects > 10 perc. pnts.  (vice-versa in brackets)  P3\_S: 14.9 (16.3)   P1\_D: 10.9 (12.5) |  |
| **P2\_M**  10 in 307 sites   3.3 %    effects > 10 perc. pnts.  (vice-versa in brackets)  P1\_F: 15.8 (12.1) |  |
| **P2\_N**  21 in 307 sites   6.8 %    effects > 10 perc. pnts.  (vice-versa in brackets)  P3\_A: 23.2 (15.8)   P1prime\_V: 22.2 (13.8) |  |
| **P2\_Q**  21 in 307 sites   6.8 %    effects > 10 perc. pnts.  (vice-versa in brackets)  P3\_I: 17.0 (17.0)   P2prime\_I: 26.7 (16.1)   P3prime\_D: 18.3 (22.6) |  |
| **P2\_S**  23 in 307 sites   7.5 %    effects > 10 perc. pnts.  (vice-versa in brackets)  P3\_I: 14.9 (16.3) |  |
